# Molecular basis of the scalp-ear-nipple syndrome unraveled by the characterization of disease-causing KCTD1 mutants

**DOI:** 10.1038/s41598-019-46911-4

**Published:** 2019-07-19

**Authors:** Giovanni Smaldone, Nicole Balasco, Luciano Pirone, Daniela Caruso, Sonia Di Gaetano, Emilia Maria Pedone, Luigi Vitagliano

**Affiliations:** 1 IRCCS SDN, Via Gianturco 113, 80143 Napoli, Italy; 2 Institute of Biostructures and Bioimaging, CNR, Via Mezzocannone 16, 80134 Napoli, Italy; 3 Università degli Studi della Campania “Luigi Vanvitelli”, Viale Abramo Lincoln 5, 81100 Caserta, Italy

**Keywords:** Biophysics, Molecular biology

## Abstract

The scalp-ear-nipple (SEN) syndrome is an autosomal-dominant disorder characterized by cutis aplasia of the scalp and malformations of breast, external ears, digits, and nails. Genetic analyses have shown that the disease is caused by missense mutations of the KCTD1 protein, although the functional/structural basis of SEN insurgence is hitherto unknown. With the aim of unravelling the molecular basis of the SEN syndrome associated with KCTD1 mutations we here expressed and characterized several disease causing mutants. A preliminary dissection of the protein provides insights into the role that individual domains play in KCTD1 stability. The characterization of SEN-causing mutants indicates that, although the mutation sites are located in distant regions of the BTB domain or of the pre-BTB region, all of them are unable to interact with the transcription factor AP-2α, a well-known KCTD1 biological partner. Notably, all mutations, including the one located in the pre-BTB region, produce a significant destabilization of the protein. The structural role of the pre-BTB region in KCTD1 and other proteins of the family is corroborated by its sequence conservation in orthologs and paralogs. Interestingly, SEN-causing mutations also favor the tendency of KCTD1 to adopt structural states that are characterized by the ability to bind the β-amyloid fluorescent dye thioflavin T. The formation of aggregation-prone species may have important implications for the disease etiology. Collectively, these findings provide an intriguing picture of the functional and structural alterations induced by KCTD1 mutations that ultimately lead to disease.

## Introduction

Members of the KCTD (Potassium Channel Tetramerization Domain) family represent an emerging class of proteins that play a key role in fundamental physio-pathological processes^1–16^. Investigations carried out in the last decade have highlighted analogies and differences among different members from both the structural and the functional points of view. Although the biochemical activities of these proteins are still somehow obscure, the biological characterizations of KCTDs have disclosed their crucial roles in highly diversified processes such as protein ubiquitination and degradation^13,15–17^, binding and modulation of the GABA_B_ receptor^18–22^, autophagy^23^, adipogenesis^24^ (*Pirone et al. unpublished results*), sleep homeostasis^25,26^ and metabolic homeostasis^27^. Not surprisingly, they are also involved in the insurgence of severe pathological states that include epilepsy, cancer, obesity, and skin diseases. A number of these pathologies are generated by mutations of KCTD proteins whose structural interpretations have been scarcely investigated^28,29^. In particular, the allelic deletion of human KCTD11 at chromosomal location 17p13.2 has been found in medulloblastoma^30,31^ whereas single nucleotide polymorphisms (SNPs) of KCTD10 (i5642G > C and V206VT > C) are associated with altered concentrations of HDL cholesterol in subjects with high levels of carbohydrate intake^9,32^. It has been also demonstrated that a missense mutation in KCTD17 (R154H) causes autosomal dominant myoclonus-dystonia (MDS)^5^. Moreover, very recently two reports independently confirm the pathogenicity of KCTD17 mutations (c.508-1G > C and c.508-2A > T splicing mutations) for MDS^33,34^. Mutants of KCTD7 (N273I, R184C, D115Y, L108M, R94W) are responsible for progressive myoclonus epilepsy^35–39^. Of particular interest is the discovery that KCTD1 genetic mutations cause the scalp-ear-nipple (SEN) syndrome (MIM 181270)^4,40^. SEN syndrome is a rare, autosomal-dominant disorder characterized by cutis aplasia of the scalp, malformations of the breast, and minor anomalies of the external ears, digits, and nails. The evaluation of a total of ten families affected by SEN syndrome revealed KCTD1 missense mutations (P20S, A30E, P31L, P31R, P31H, H33Q, H33P, G62D, D69E, and H74P) in each family tested although the functional and structural basis of the insurgence of the disease are unknown.

Independent literature studies have shown the involvement of KCTD1 in distinct biological processes. Indeed, it has been shown that this protein works as a potent transcriptional repressor of AP-2α transcriptional activity and as a suppressor of the canonical Wnt signaling pathway by enhancing β-catenin degradation^41^. We have very recently shown that KCTD1 is also involved in adipogenesis. Moreover, *kctd1* is target gene of miR-155-3p that regulates TNF-α-inhibited osteogenic differentiation^42^. From the molecular point of view, KCTD1 is constituted by two distinct domains: a BTB domain located at the N-terminal region of the protein (approximately residues 30–135) and a putative C-terminal domain (approximately residues 136–257)^9,24,43–45^. Moreover, an N-terminal tail whose sequence exhibits a high number of proline residues precedes the BTB domain.

With the aim of unravelling the molecular basis of the SEN syndrome associated with KCTD1 mutations we here expressed and characterized several disease causing mutants. Our analyses clearly indicate that these mutations, although located in distant regions of the BTB domain or of the pre-BTB region, impair the KCTD1 ability to interact with AP-2α. Interestingly, these mutations also favor the tendency of KCTD1 to adopt structural states that are characterized by the ability to bind the β-amyloid fluorescent dye thioflavin T (ThT). Collectively, these findings provide a clear picture of the functional and structural alterations of the protein induced by the mutations that ultimately lead to the disease.

## Results

### Characterization of KCTD1 and its individual domains

**Figure 1:**
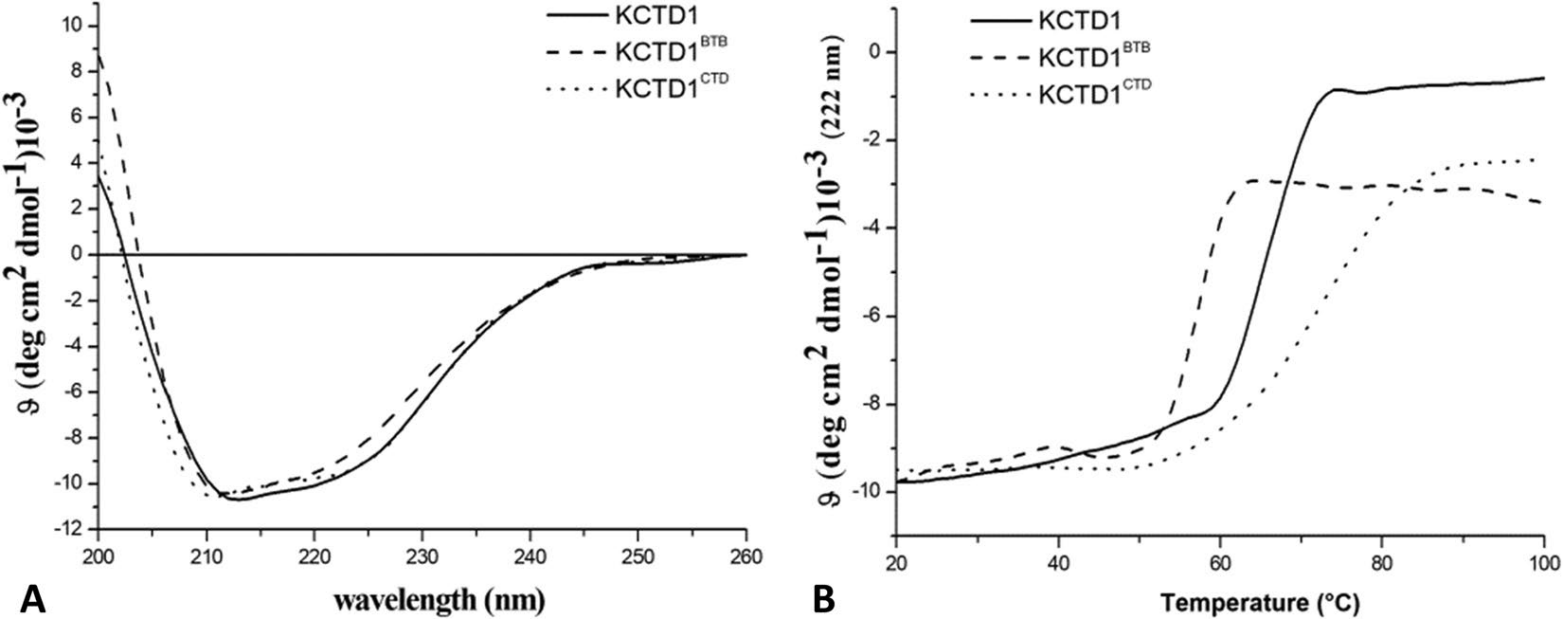
Biophysical characterization of KCTD1, KCTD1^BTB^, and KCTD1^CTD^: Far-UV CD spectra recorded at 20 °C (**A**), thermal denaturation curves obtained by monitoring the CD signal at 222 nm in the temperature range 20–100 °C (**B**).

KCTD1, KCTD1^BTB^, and KCTD1^CTD^ were recombinantly expressed and purified with a high level of homogeneity and in good yields as reported in the Methods section. The Circular Dichroism (CD) spectra of KCTD1 and its individual domains are indicative of well-folded α/β proteins (Fig. 1A), in line with the secondary structure predictions (Fig. S1). The analysis of the evolution of the CD signal as function of temperature indicates that all these protein variants are endowed with a good thermal stability (Fig. 1B and Table 1). Thermal denaturation experiments show that the full length protein presents a melting temperature of 64 °C. Notably, KCTD1^BTB^ displays a reduced stability as its denaturation profile is suggestive of a Tm value of 57 °C. Surprisingly, the thermal denaturation analysis of KCTD1^CTD^ evidences that this domain is endowed with a remarkable stability (Tm of 73 °C) and it is more stable than both the BTB domain and the entire protein. These findings demonstrate that the overall stability of the full length protein relies more on its C-terminal domain than on the BTB that is typically assumed to be an oligomerization-promoting domain.

**Table 1:** Melting temperatures Tm of KCTD1 proteins obtained from CD data.

Protein	Tm (°C)
KCTD1	67
KCTD1^BTB^	57
KCTD1^CTD^	74
KCTD1^P20S^	62
KCTD1^H33P^	62
KCTD1^G62D^	61
KCTD1^D69E^	61
KCTD1^H74P^	60

**Figure 2:**
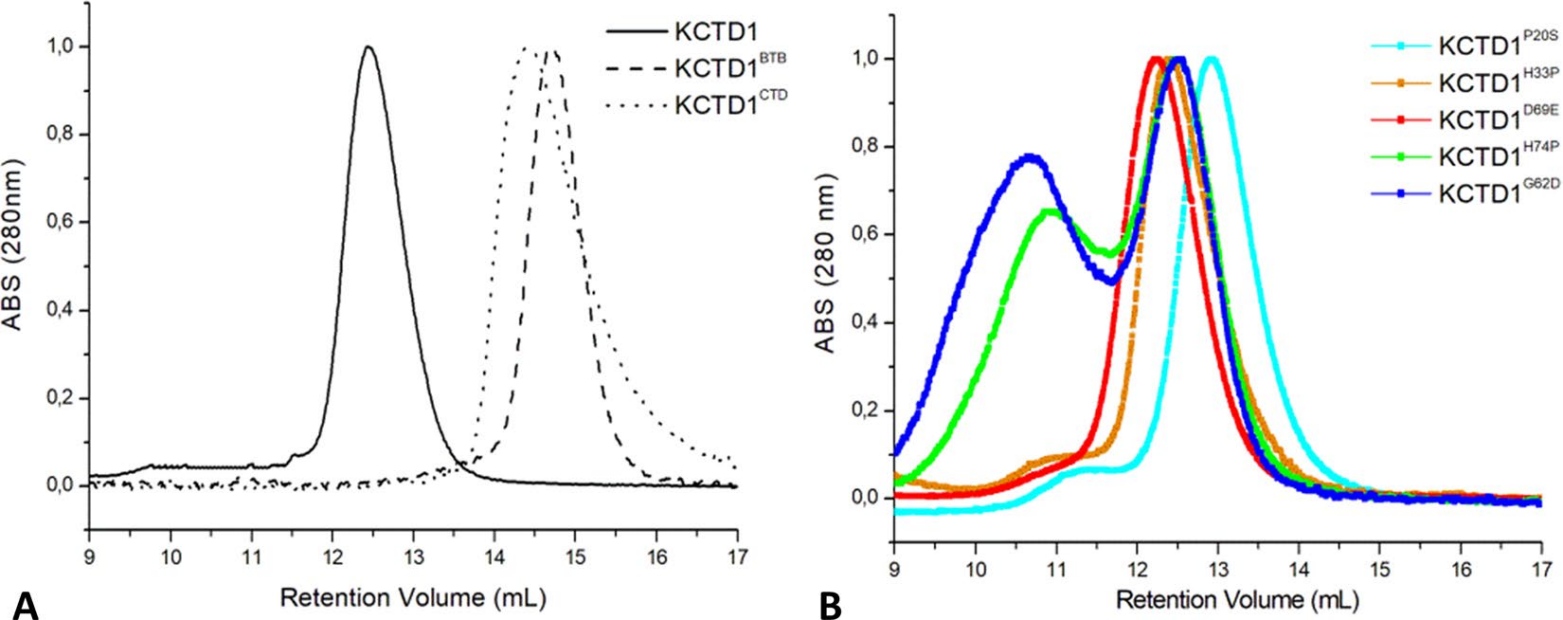
Gel filtration elution profiles of KCTD1, KCTD1^BTB^, KCTD1^CTD^ (**A**) and of the SEN-causing mutants KCTD1^P20S^, KCTD1^H33P^, KCTD1^G62D^, KCTD1^D69E^, KCTD1^H74P^ (**B**). The MW markers used for reference are: blue dextran 2000 (Mw > 1000 kDa, retention volume 7.8 mL), ovalbumin (Mw 43 kDa, retention volume 14.9 mL), carbonic anhydrase (Mw 29 kDa, retention volume 15.6 mL), and ribonuclease A (Mw 13.7 kDa, retention volume 17.2 mL).

The estimation of the molecular weights of the three variants was performed by analytical size-exclusion chromatography (SEC) analyses (see Methods). As shown in Fig. 2A, the elution volumes of KCTD1, KCTD1^BTB^, and KCTD1^CTD^ were 12.5, 14.6, and 14.5 mL, respectively. Taking into account the apparent molecular weights of the recombinant proteins and the theoretical molecular weights of the protein monomers, the number of the chains for the three variants are ~4.5 (Table S1). This value is in good agreement with the functional pentameric state of KCTD proteins^43,44,46^. The ability of KCTD1^CTD^ to form highly stable oligomeric states suggests that in KCTD1 the BTB domain does not play the expected role of oligomerization domain.

### Characterization of SEN-causing KCTD1 variants

#### Localization and selection of representative mutants

**Figure 3:**
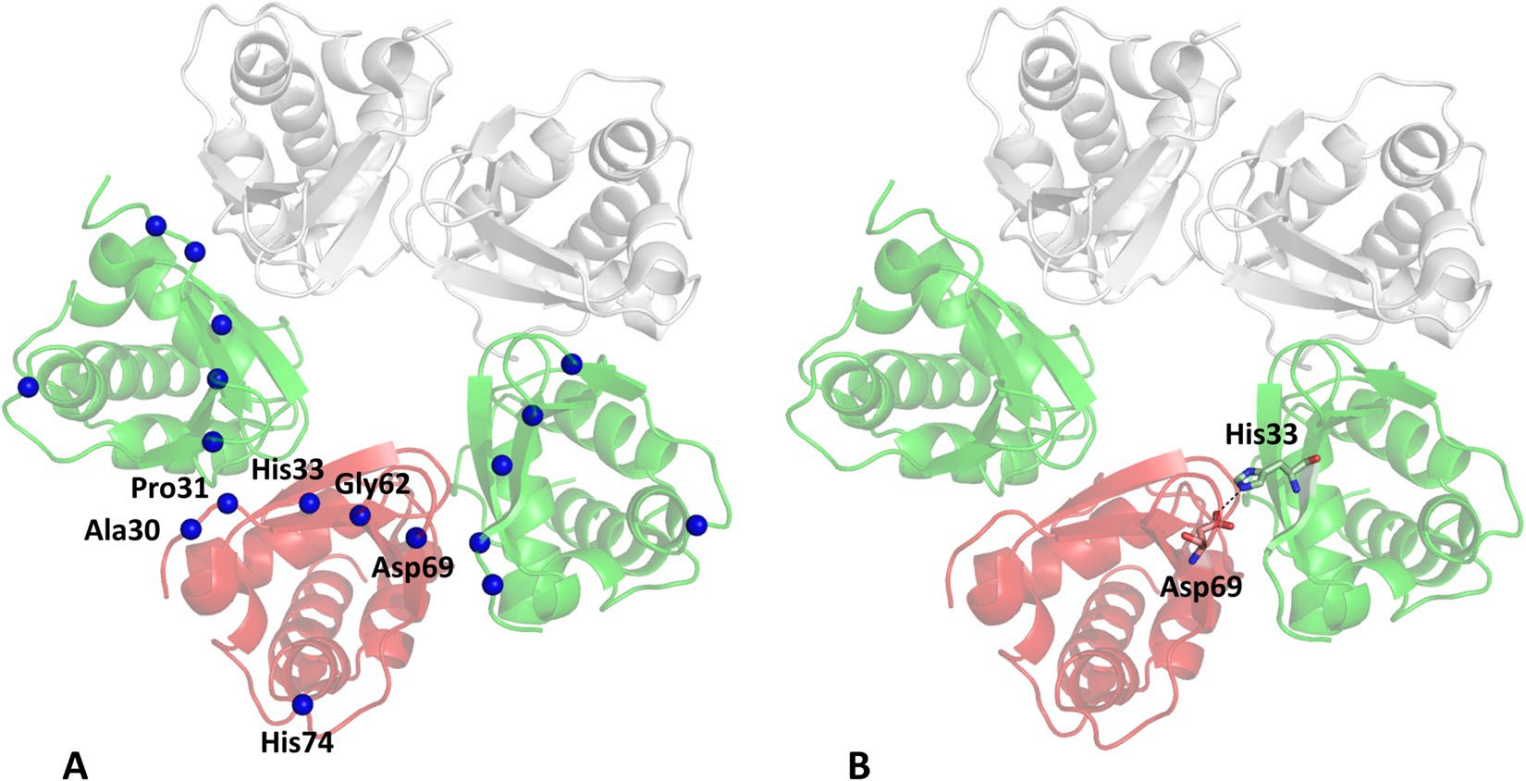
Localization in KCTD1^BTB^ structure (PDB ID: 5BXB) of the amino acid residues (Ala30, Pro31, His33, Gly62, Asp69, and His74) that are mutated in SEN-causing single-point KCTD1 mutants (**A**). Salt bridge formed by the side chains of residues His33 and Asp69 of adjacent KCTD1^BTB^ chains (**B**).

As previously mentioned, the SEN syndrome is caused by ten unique missense KCTD1 mutations (P20S, A30E, P31L, P31R, P31H, H33Q, H33P, G62D, D69E, and H74P) that are localized in the N-terminal region of the protein. Indeed nine of them are located in the BTB domain whereas the P20S mutation is embodied in the pre-BTB region of the protein^4^. The recent determination of the crystal structure of KCTD1^BTB^^44^ (PDB ID: 5BXB) that covers the region 28–132 provided us the possibility to analyze the structural context of the nine mutations falling in the BTB domain and to gain insights into the role that they play to maintain the structural integrity of the protein. Therefore, we performed molecular modeling analyses on the possible impact that SEN-causing KCTD1 mutations may have on the protein structure. Interestingly, although some of them are distant in the sequence, they are spatially clustered as shown in Fig. 3A. For eight out of ten SEN related mutations, a destabilization of the structural integrity of the protein, though different mechanisms, is suggested. In particular, Pro31 side chain is tightly packed with the rest of the protein. Therefore, its replacement with bulkier residues (Pro31Leu, Pro31His, and Pro31Arg) may produce destabilizing steric clashes. Notably, the side chains of two mutated residues (His33 and Asp69) form a tight salt bridge that would be destabilized in His33Gln and His33Pro (Fig. 3B**)**. The destabilizing effects of the conservative mutation Asp69Glu cannot be easily explained, unless steric effects caused by the bulkier Glu side chain are invoked. The mutation Gly62Glu may destabilize the protein structure as in the native protein the Gly residue adopts dihedral angles (ϕ, ψ) ~ (95°, 0°) that are disfavored for non-Gly residues. Similarly, the His74Pro mutant may also be unstable since the proline cannot adopt the conformation assumed by the histidine in the protein structure (ϕ, ψ) ~ (−150°, 150°). Somewhat puzzling is the analysis of the impact of the other two mutations on the protein structure. As Ala30 is exposed, its replacement with a glutamic acid residue should not produce drastic effects. Even more surprising is the relation between SEN and the Pro20Ser mutation as this residue is located in a region that should be disordered according to PSIpred^47^ (Fig. S1A). Notably, secondary structure predictions carried out using the s2D method^48^, which extends the predictive analysis to the polyproline II motif, highlight that the pre-BTB region of the protein has some tendency to adopt this type of structure (Fig. S1B).

#### Characterization of representative SEN-causing KCTD1 mutants and their ability to bind Thioflavin T

In order to relate the pathological state with the functional and/or structural perturbations caused by KCTD1 mutations, single-point mutants of the protein were expressed and purified to homogeneity with good yields. Specifically, on the basis of the considerations reported in the previous paragraph, we focused our attention on five mutants (KCTD^P20S^, KCTD^H33P^, KCTD^G62D^, KCTD^D69E^, and KCTD^H74P^) as representative examples of SEN-causing mutations as likely destabilize the native protein using different mechanisms and as they generate severe phenotypes^4^. We initially evaluated the apparent oligomerization state of these variants by SEC analysis (Fig. 2B). These experiments indicate that most of these variants present elution times, which fall in the range 12.2–12.9 mL, similar to that exhibited by the wild-type protein (Fig. 2). This finding suggests that the destabilizations induced by the mutation do not alter their propensity to oligomerize. It is worth mentioning that a second large peak at lower retention volumes that is likely associated to larger assemblies is observed for two of these variants (KCTD1^G62D^ and KCTD1^H74P^) and, at much lower extent, for the other mutants (Fig. 2B).

**Figure 4:**
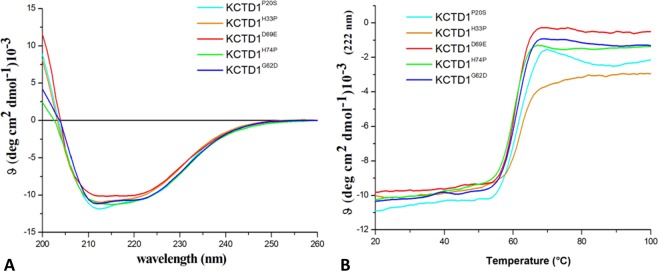
Biophysical characterization of SEN-causing KCTD1 mutants: KCTD1^P20S^, KCTD1^H33P^, KCTD1^G62D^, KCTD1^D69E^, KCTD1^H74P^. Far-UV CD spectra recorded at 20 °C (**A**), thermal denaturation curves obtained by monitoring the CD signal at 222 nm in the temperature range 20–100 °C (**B**).

CD spectra indicate that all KCTD variants are folded although some differences in the spectra could be detected (Fig. 4A). These changes in the CD spectra are similar to those induced by the addition of Cu^2+^ ions to KCTD1 samples^49^. Thermal denaturation analyses show that all mutations significantly destabilize the protein structure (Fig. 4B and Table 1). Indeed, the Tm values exhibited by the mutants are 5–7 °C lower than those shown by the wild-type protein.

**Figure 5:**
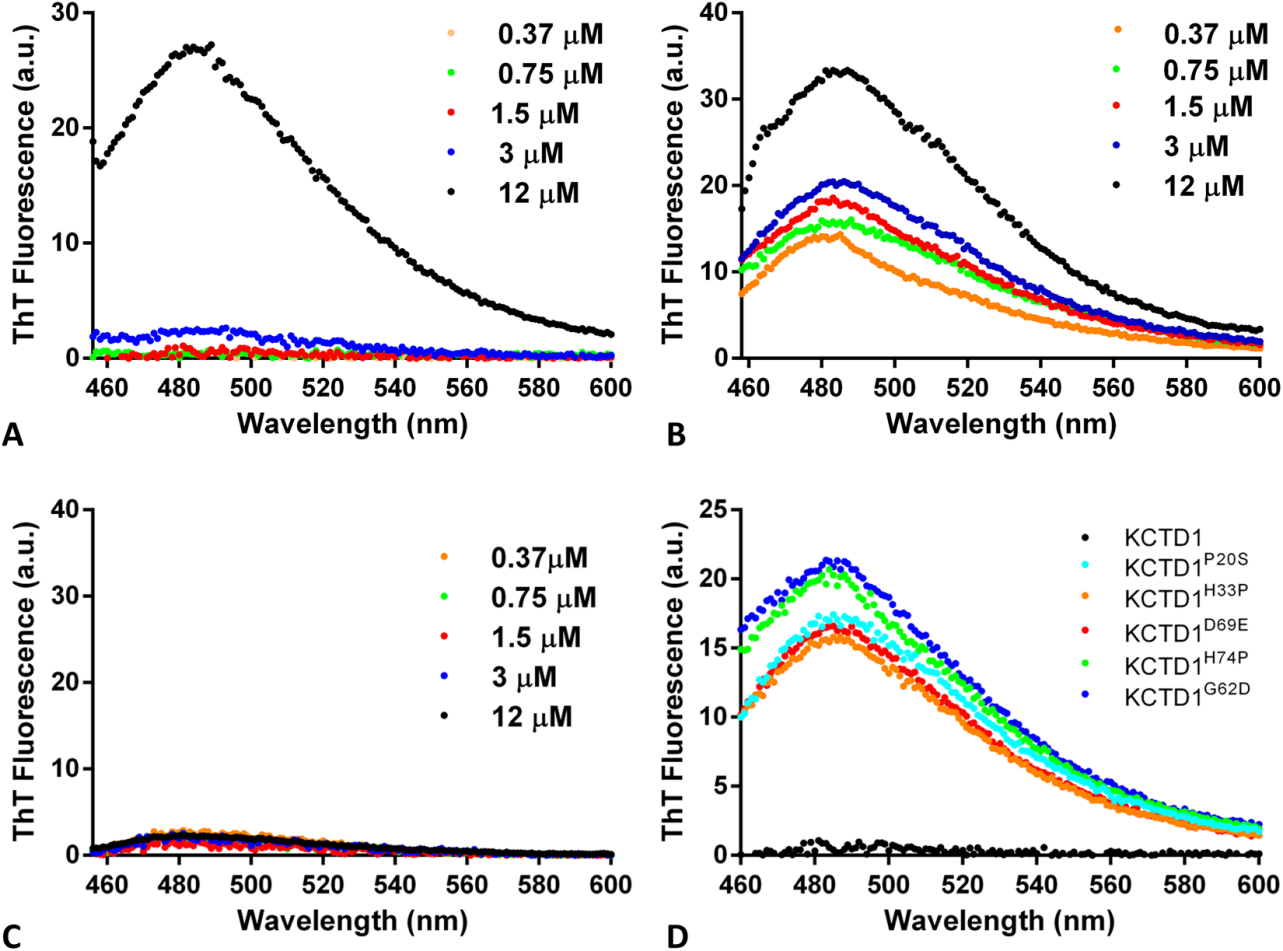
Thioflavin T Fluorescence assay: emission spectra obtained by adding a ThT solution (50 μM) to KCTD1 (**A**), KCTD1^BTB^ (**B**), KCTD1^CTD^ (**C**) at protein concentrations of 0.37 μM, 0.75 μM, 1.5 μM, 3 μM, and 12 μM, and to KCTD1^P20S^, KCTD1^H33P^, KCTD1^G62D^, KCTD1^D69E^, KCTD1^H74P^ (**D**) at protein concentration of 1.5 μM.

It has been recently reported that the copper ion is able to destabilize the protein structure and generate KCTD1 amyloid-like and toxic aggregates that are able to bind the ThT dye^49^. ThT is a benzothiazole dye that exhibits enhanced fluorescence with a peak at ~482 nm after excitation in the 430–450 nm region upon binding to amyloid-like aggregates^50^. In this scenario, we evaluated the propensity of the different KCTD1 variants here expressed to bind this compound that is universally believed to be an effective probe of amyloid-like states. In particular, a solution of ThT was added to KCTD1 proteins at different concentrations (ranging from 0.37 μM to 12 μM). As shown in Fig. 5A, the full-length protein tends to bind the dye only at the highest concentration tested (12 μM). The BTB portion of the protein does bind the dye even at low concentrations (Fig. 5B) whereas the C-terminal domain does not anchor ThT (Fig. 5C). This result is likely due to the destabilization of the domain caused by the removal of the very stable C-terminal domain. This finding is in line with the observation that the isolated KCTD1^BTB^ presents heterogeneous structural states^44,46^. As shown in Figs S2 and 5D, the different SEN-causing mutants display a propensity to bind the dye. Interestingly, these data indicate that the mutations favor the formation of aggregation-prone KCTD1 variants.

#### Binding of KCTD1 and its SEN-causing variants to AP-2α^NTD^

**Figure 6:**
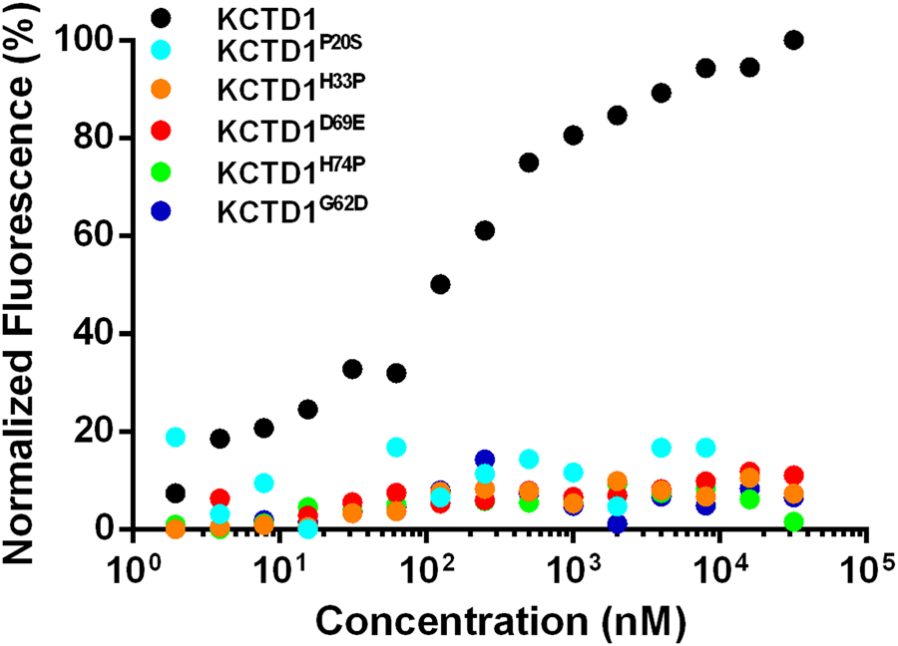
Quantification of the binding of KCTD1 and its SEN related mutants KCTD1^P20S^, KCTD1^H33P^, KCTD1^G62D^, KCTD1^D69E^, KCTD1^H74P^ to AP-2α^NTD^ by Microscale Thermophoresis experiments.

**Figure 7:**
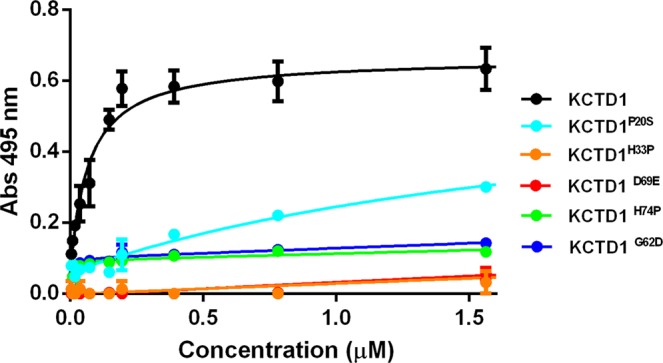
Binding of KCTD1 and its SEN related mutants KCTD1^P20S^, KCTD1^H33P^, KCTD1^G62D^, KCTD1^D69E^, KCTD1^H74P^ to AP-2α^NTD^ by ELISA assays.

**Figure 8:**
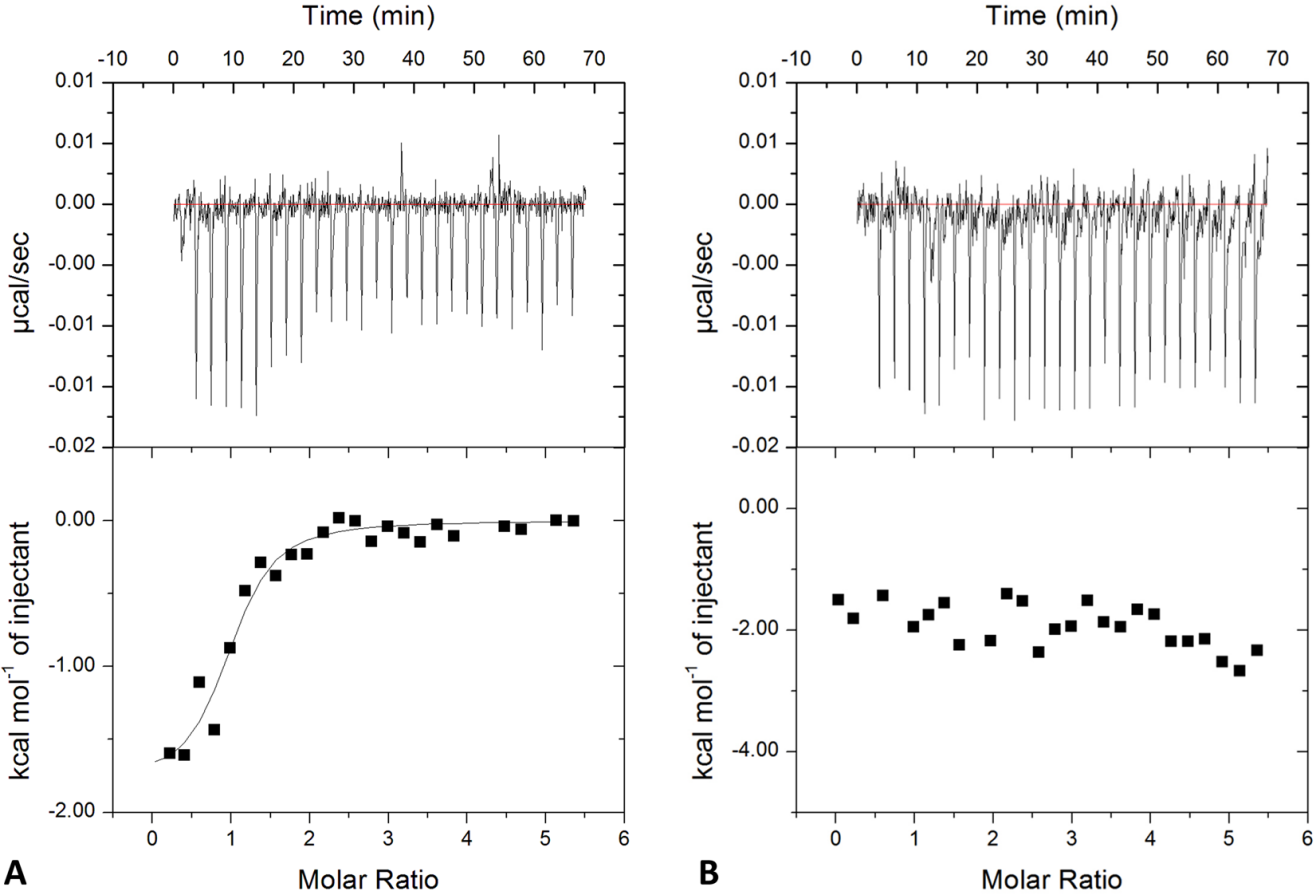
Quantification of KCTD1 binding to AP-2α^NTD^ by Isothermal Titration Calorimetry. ITC experiments were performed by titrating KCTD1 (72 μM-expressed as pentamer) into a solution of AP-2α^NTD^ (7 μM) (**A**) or a buffer solution (**B**). Top and bottom panels report raw and integrated data, respectively.

Literature data suggest that KCTD1 is able to (negatively) modulate the transcription activity of AP-2α^41^. In order to correlate the syndrome with the ability/inability of KCTD1 SEN-related mutants to bind the transcription factor we have preliminary expressed and characterized the N-terminal region of AP-2α (residues 1–165) (see Methods for details). In line with previous results^51^, the CD spectroscopic data of AP-2α^NTD^ confirms that this region of the protein is natively unfolded (data not shown). The binding of AP-2α^NTD^ to the KCTD1 variants was initially evaluated by microscale thermophoresis. Although we were unable to collect enough points in the saturation region due to the low solubility of the proteins, the titration curves of KCTD1 and AP-2α^NTD^ qualitatively indicate that the wild-type KCTD1 binds AP-2α^NTD^ whereas all mutants do not (Fig. 6). This observation was corroborated and quantified by ELISA assays. As reported in Fig. 7, KCTD1 displays a good affinity for AP-2α^NTD^ with an apparent binding constant in the sub μM range (Kd = 593 ± 69 nM). Again, all SEN-related mutants do not show any appreciable binding to AP-2α^NTD^. In order to confirm and quantify these data, isothermal titration calorimetry (ITC) experiments were also performed (Fig. 8). The negligible heat exchanges associated with the titration of KCTD1 SEN related mutants with AP-2α^NTD^ suggest that no binding occurs (Fig. S3). This observation is in line with the ELISA and thermophoresis results previously described. On the other hand, measurable heat exchanges were observed for the wild-type KCTD1 (Fig. 8A). The quantification of the binding yielded a value for the dissociation constant Kd of 850 ± 115 nM (ΔH = −20.2 ± 0.1 kcal/mol). The value of the number of sites of KCTD1 *per* AP-2α^NTD^ is ~1 suggesting that a pentamer of KCTD1 binds a single molecule of AP-2α^NTD^.

## Discussion

Investigations carried out in the last decade have delineated the crucial role of KCTD proteins in several physio-pathological processes. In most of the cases, however, these findings could not be related to the biochemical and structural properties of these proteins due to the paucity of available data. Fortunately, this scenario is rapidly changing. Indeed, structural characterizations of these proteins have made significant progresses in the last three years^14,43,44^. The determination of the structure of several BTB domains of KCTD proteins has highlighted an impressive structural versatility of these domains in terms of oligomeric states and of open/closed states. The finding that these domains generally form closed pentameric states when anchored to their functional partner cullin 3 suggested that the variability of diverse oligomeric states exhibited by the BTB domains was an artifact generated by the deletion of the C-terminal regions of the proteins. However, the recent characterization of the KCTD12 BTB domain shows that it anchors the GABA_B_ receptor in an open pentameric form thus suggesting that the structural variability of these domains is, at least in part, functionally relevant^14^. With the aim of gaining new functional/structural information on this important class of proteins, we here explored the mechanism underlying the insurgence of the SEN syndrome induced by KCTD1 mutations. We preliminary dissected the protein structure into its constituent domains (BTB and CTD). The characterization of these domains indicates that both of them are able to individually form oligomeric assemblies. Interestingly, the CTD domain is more stable than the BTB one, thus providing the strongest contribution to KCTD1 overall stability. These observations suggest that functional studies should be ideally carried out considering the entire protein. Accordingly, we investigated the impact of SEN-causing mutations by considering the framework of the full-length KCTD1. Functional studies show that all KCTD1 mutants here considered lose the ability to bind AP-2α^NTD^, the best characterized KCTD1 partner. Interestingly, as previously underlined^4^, mutations of AP-2α cause cutis aplasia in individuals with an analogous syndrome, the branchio-oculo-facial (BOFS-MIM 113620)^52^. Collectively, these observations provided a strong link between the functional impairment of the protein and the disease.

Interestingly, although the mutations here characterized involve residues with quite different physico-chemical properties, they produce similar destabilizations of the protein. An inspection of the KCTD1^BTB^ three-dimensional structure (PDB ID: 5BXB) provides rather straightforward explanations to the destabilizing effects caused by the mutations H33P, G62D, and H74P. The destabilizations caused by the other two mutations D69E and P20S are more difficult to interpret. The destabilization induced by the conservative mutation D69E suggests that even a small modification of the side chains impairs the correct formation of the salt bridge formed by this residue with H33. The observed destabilization caused by this mutation highlights the remarkable structural susceptibility of the protein. Even more puzzling is the observation that the mutations P20S, which is located in the pre-BTB region of the protein, produces destabilizing effects comparable to those induced by mutations located in the core of the BTB structure. This finding strongly suggests that the pre-BTB region whose role has been generally overlooked in KCTD-focused studies plays an active role in the stabilization of the protein. Predictive analysis and the inspection of the pre-BTB sequence (Fig. S4) suggest that this fragment may adopt a polyproline structure. The role of the pre-BTB region in the protein stabilization is corroborated by comparative sequence analyses among KCTD1 orthologs and paralogs. The sequence of this region is well conserved in the KCTD15 paralog (Fig. S4), where also the residue Pro20 is preserved. It is worth mentioning that the conservation of the pre-BTB region is not a common feature among KCTD evolutionary clades (Fig. S4). Although this portion of the protein generally presents an abundance of Pro residues, in several cases its sequence is not conserved among paralogs (Fig. S4). It is likely that the sequence conservation is associated with a possible structural/functional role of pre-BTB. The pre-BTB region is well conserved also in KCTD1 orthologs. As shown in Table S2, this sequence is conserved in species representing different classes of the Chordata phylum or Nematoda phylum. In all cases the residue Pro20 is also conserved. Notably, the conservation of the pre-BTB sequence among KCTD orthologs is not a widespread property being observed only for KCTD1/KCTD15, KCTD6, KCTD9 and KCTD10 (data not shown). It is worth mentioning that, in general, KCTD1 is highly conserved during evolution as its sequence has been virtually unchanged over the last 65 million years. Indeed, the sequences of the human and mouse KCTD1 counterparts are identical, also considering the pre-BTB region. Similarly, human and rat sequences present 256 strictly conserved residues out of 257.

One intriguing properties of these SEN-causing mutants is their ability to interact with ThT, a universally used probe of the amyloid state. The tendency of KCTD1 to form amyloid-like and toxic aggregates has been recently discovered in conditions enriched of the ion Cu^2+^^49^. The structural transition toward aggregation-prone states of the SEN-causing mutants may contribute to the functional impairment of the protein. It can be speculated that the amyloid-like aggregation of KCTD1 mutants may contribute to the sequestration of the active form of the protein in an inactive state. In line with the proposal made for the prion-like behavior of oncogenic p53 mutants^53–55^, this observation may explain the dominance effect of SEN mutations. Amyloid-like aggregation in neurodegeneration is a hallmark event whereas its involvement in other pathologies has been sporadically reported^56,57^. The marginal involvement of this type of aggregation in the etiology of diseases is somehow paradoxal since the formation of β-rich aggregates is a primordial and widespread phenomenon of the polypeptide chain. In this scenario, present finding suggests a potential novel example of the role that aggregation may play in contexts that go beyond neurodegeneration.

## Materials and Methods

### Cloning, expression and purification of KCTD1 and its mutants

Genes encoding the full-length KCTD1 protein (residues 1–257) and its BTB domain (KCTD1^BTB^, residues 1–135) were purchased from Sigma Aldrich (Milan, Italy). Single point mutants of the full-length KCTD1 (KCTD1^P20S^, KCTD1^H33P^, KCTD1^D69E^, KCTD1^H74P^, KCTD1^G62D^) were obtained by site-specific mutagenesis reactions using QuikChange mutagenesis kit (Stratagene, Basel, Switzerland.) (Table S3). The truncated protein variant corresponding to the C-terminal domain of the protein (KCTD1^CTD^, residues 134–235) was generated by PCR reaction. All proteins were cloned into pETM11 expression vector with an N-terminal polyHis tag that is removable by TEV protease. KCTD1, KCTD1^BTB^, and single point mutants were expressed *in Escherichia coli* BL21(DE3) cells inducing protein expression through the addition of IPTG 0.5 mM at 16 °C for 16 h and purified as previously reported^46^. The expression and purification of the N-terminal domain of AP-2α (AP-2α^NTD^, residues 1–165) followed the procedures reported elsewhere^51^. After his-trap purification, KCTD1, KCTD1^BTB^ and the single point mutants were digested with TEV protease to remove His-Tag. All protein variants were loaded on a Superdex 200 10/30 (GE Healthcare, Uppsala, Sweden) column previously equilibrated in a buffer containing Tris-HCl 20 mM (pH 7.8), NaCl 100 mM, Glycerol 5%, CHAPS 0.01%, and DTT 4 mM. The estimation of the molecular weights of the KCTD1 variants was performed by gel filtration upon column calibration with the Gel Filtration calibration kit (GE Healthcare). The markers contained in this kit are blue dextran 2000 (Mw > 1000 kDa, elution volume7.8 mL), ovalbumin (Mw 43 kDa, elution volume 14.9 mL), carbonic anhydrase (Mw 29 kDa, elution volume 15.6 mL), and ribonuclease A (Mw 13.7 kDa, elution volume17.2 mL).

Cells expressing AP-2α^NTD^ were resuspended in a buffer containing Tris-HCl 50 mM (pH 8), NaCl 500 mM, and urea 8 M and sonicated for 10 minutes. The soluble fraction was recovered by centrifugation at 17,000 rpm for 30 minutes and HisTrap purification on batch using Ni-NTA resin (Qiagen, Milan, Italy) was performed. Fractions containing the protein have been extensively dialyzed against H_2_O to remove salts. The pellet was dissolved in CH_3_CN 20% and TFA 0.1% and loaded on a HPLC *proteo* column (Jupiter, 250 × 10 mm, Phenomenex) pre-equilibrated in the same buffer for final step of purification. The purified protein has been lyophilized and stored at −20 °C.

### Circular dichroism experiments

The correct folding of all proteins was assessed by Far-UV CD spectroscopy. The spectra were recorded in the 190–260-nm range on a Jasco J-710 spectro polarimeter equipped with a Peltier thermostatic cell holder (Jasco Europe, Cremella, LC, Italy model PTC-343). The spectra were registered under a constant N_2_ flow carried out at 20 °C using the following experimental parameters: scanning speed of 20 nm/min, band width of 2 nm, and response time of 4 s. Spectra were recorded using a protein concentration of 2 µM for KCTD1 and 4 µM for KCTD1^BTB^ and the mutants. Thermal denaturation curves of KCTD1 and its single point mutants were recorded over the 20–100 °C temperature interval monitoring the CD signal at 222 nm. The curves were registered using a 0.1 cm path length cell and a scan rate of 1.0 °C/min.

### Binding assays (ELISA, ITC and Thermophoresis)

ELISA assays were performed using 3 µM AP-2α^NTD^ in coating and increasing concentrations of KCTD1 and its single point mutants (from 1.8 nM to 1.8 µM). Anti KCTD1 antibody (ab104012, abcam, Cambridge, UK) was used for the detection.

ITC studies were performed at 22 °C with an ITC200 calorimeter (MicroCal/GE Healthcare, Milan, Italy). KCTD1 and its mutants (at concentration of 72 μM expressed as pentamer) were titrated into a solution of AP-2α^NTD^ (7 μM). Fitting of data to a single-binding site model was performed with the Origin software as supplied by GE Healthcare. ITC runs were repeated twice to evaluate the reproducibility of the results.

Microscale Thermophoresis experiments were performed on a Monolith NT 115 system (Nano Temper Technologies, Munchen, Germany) using 100% LED and 20% IR-laser power. The labelling of AP-2α^NTD^ (20 µM) was performed with NT-647 reactive dye (provided by Nano temper), which reacts efficiently with the primary amines of the proteins to form stable dye-protein conjugates. A 16-point serial dilution (1:1) was prepared for KCTD1 and its mutants at final concentrations ranging from 0.6 nM to 20 µM. The samples were filled into Premium capillaries.

### ThT fluorescence experiments

ThT solution (50 μM) was added to different concentrations of KCTD1 proteins (from 0.37 μM to 12 μM). ThT fluorescence was measured with a Varian Cary Eclipse spectrofluorimeter (Agilent Technologies, Stamford, CT, USA) and a quartz cuvette cell of 10 mm path-length. Measurements were collected at 25 °C with excitation and emission wavelengths of 440 and 450–600 nm, respectively.

## Supplementary information

Supplementary Material

## References

[1] De Smaele E (2011). Identification and characterization of KCASH2 and KCASH3, 2 novel Cullin3 adaptors suppressing histone deacetylase and Hedgehog activity in medulloblastoma. Neoplasia.

[2] Azizieh R (2011). Progressive myoclonic epilepsy-associated gene KCTD7 is a regulator of potassium conductance in neurons. Mol Neurobiol.

[3] Golzio C (2012). KCTD13 is a major driver of mirrored neuroanatomical phenotypes of the 16p11.2 copy number variant. Nature.

[4] Marneros AG (2013). Mutations in KCTD1 cause scalp-ear-nipple syndrome. Am J Hum Genet.

[5] Mencacci NE (2015). A missense mutation in KCTD17 causes autosomal dominant myoclonus-dystonia. Am J Hum Genet.

[6] Schwenk J (2016). Modular composition and dynamics of native GABAB receptors identified by high-resolution proteomics. Nat Neurosci.

[7] Correale S (2011). Molecular organization of the cullin E3 ligase adaptor KCTD11. Biochimie.

[8] Kasahara K (2014). Ubiquitin-proteasome system controls ciliogenesis at the initial step of axoneme extension. Nat Commun.

[9] Liu Z, Xiang Y, Sun G (2013). The KCTD family of proteins: structure, function, disease relevance. Cell Biosci.

[10] Stogios PJ, Downs GS, Jauhal JJ, Nandra SK, Prive GG (2005). Sequence and structural analysis of BTB domain proteins. Genome Biol.

[11] Escamilla CO (2017). Kctd13 deletion reduces synaptic transmission via increased RhoA. Nature.

[12] Brockmann M (2017). Genetic wiring maps of single-cell protein states reveal an off-switch for GPCR signalling. Nature.

[13] Canettieri G (2010). Histone deacetylase and Cullin3-REN(KCTD11) ubiquitin ligase interplay regulates Hedgehog signalling through Gli acetylation. Nat Cell Biol.

[14] Zheng S, Abreu N, Levitz J, Kruse AC (2019). Structural basis for KCTD-mediated rapid desensitization of GABAB signalling. Nature.

[15] Smaldone G (2015). Cullin 3 Recognition Is Not a Universal Property among KCTD Proteins. PLoS One.

[16] Balasco N (2014). Molecular recognition of Cullin3 by KCTDs: insights from experimental and computational investigations. Biochim Biophys Acta.

[17] Chen Y (2009). Cullin mediates degradation of RhoA through evolutionarily conserved BTB adaptors to control actin cytoskeleton structure and cell movement. Mol Cell.

[18] Schwenk J (2010). Native GABA(B) receptors are heteromultimers with a family of auxiliary subunits. Nature.

[19] Adelfinger L (2014). GABAB receptor phosphorylation regulates KCTD12-induced K(+) current desensitization. Biochem Pharmacol.

[20] Turecek R (2014). Auxiliary GABAB receptor subunits uncouple G protein betagamma subunits from effector channels to induce desensitization. Neuron.

[21] Pirone L (2011). Design, synthesis and characterization of a peptide able to bind proteins of the KCTD family: implications for KCTD-cullin 3 recognition. J Pept Sci.

[22] Correale S (2013). A biophysical characterization of the folded domains of KCTD12: insights into interaction with the GABAB2 receptor. J Mol Recognit.

[23] Teng X, Hardwick JM (2019). Whi2: a new player in amino acid sensing. Curr Genet.

[24] Skoblov M (2013). Protein partners of KCTD proteins provide insights about their functional roles in cell differentiation and vertebrate development. Bioessays.

[25] Pfeiffenberger C, Allada R (2012). Cul3 and the BTB adaptor insomniac are key regulators of sleep homeostasis and a dopamine arousal pathway in Drosophila. PLoS Genet.

[26] Pirone L (2016). Proteins involved in sleep homeostasis: Biophysical characterization of INC and its partners. Biochimie.

[27] Williams MJ (2014). Obesity-linked homologues TfAP-2 and Twz establish meal frequency in Drosophila melanogaster. PLoS Genet.

[28] Barone D, Balasco N, Vitagliano L (2016). KCTD5 is endowed with large, functionally relevant, interdomain motions. J Biomol Struct Dyn.

[29] Moen MN (2016). Pathogenic variants in KCTD7 perturb neuronal K+ fluxes and glutamine transport. Brain.

[30] De Smaele E (2004). Chromosome 17p deletion in human medulloblastoma: a missing checkpoint in the Hedgehog pathway. Cell Cycle.

[31] Di Marcotullio L (2004). REN(KCTD11) is a suppressor of Hedgehog signaling and is deleted in human medulloblastoma. Proc Natl Acad Sci USA.

[32] Junyent M (2009). Novel variants at KCTD10, MVK, and MMAB genes interact with dietary carbohydrates to modulate HDL-cholesterol concentrations in the Genetics of Lipid Lowering Drugs and Diet Network Study. Am J Clin Nutr.

[33] Marcé-Grau Anna, Correa Marta, Vanegas Maria Isabel, Muñoz-Ruiz Teresa, Ferrer-Aparicio Silvia, Baide Heidy, Macaya Alfons, Pérez-Dueñas Belén (2019). Childhood onset progressive myoclonic dystonia due to a de novo KCTD17 splicing mutation. Parkinsonism & Related Disorders.

[34] Graziola Federica, Stregapede Fabrizia, Travaglini Lorena, Garone Giacomo, Verardo Margherita, Bosco Luca, Pro Stefano, Bertini Enrico, Curatolo Paolo, Vigevano Federico, Capuano Alessandro (2019). A novel KCTD17 mutation is associated with childhood early-onset hyperkinetic movement disorder. Parkinsonism & Related Disorders.

[35] Van Bogaert P (2007). Mutation of a potassium channel-related gene in progressive myoclonic epilepsy. Ann Neurol.

[36] Van Bogaert P (2016). KCTD7-related progressive myoclonus epilepsy. Epileptic Disord.

[37] Staropoli JF (2012). A Homozygous Mutation in KCTD7 Links Neuronal Ceroid Lipofuscinosis to the Ubiquitin-Proteasome System. Am J Hum Genet.

[38] Kousi M (2012). Novel mutations consolidate KCTD7 as a progressive myoclonus epilepsy gene. J Med Genet.

[39] Krabichler B (2012). Novel Mutation in Potassium Channel related Gene KCTD7 and Progressive Myoclonic Epilepsy. Ann Hum Genet.

[40] Finlay AY, Marks R (1978). An hereditary syndrome of lumpy scalp, odd ears and rudimentary nipples. Br J Dermatol.

[41] Ding X (2009). The interaction of KCTD1 with transcription factor AP-2alpha inhibits its transactivation. J Cell Biochem.

[42] Wang X (2017). MicroRNA-155-3p Mediates TNF-alpha-Inhibited Cementoblast Differentiation. J Dent Res.

[43] Pinkas DM (2017). Structural complexity in the KCTD family of Cullin3-dependent E3 ubiquitin ligases. Biochem J.

[44] Ji AX (2016). Structural Insights into KCTD Protein Assembly and Cullin3 Recognition. J Mol Biol.

[45] Dementieva IS (2009). Pentameric assembly of potassium channel tetramerization domain-containing protein 5. J Mol Biol.

[46] Smaldone G (2016). The BTB domains of the potassium channel tetramerization domain proteins prevalently assume pentameric states. FEBS Lett.

[47] Jones DT (1999). Protein secondary structure prediction based on position-specific scoring matrices. J Mol Biol.

[48] Sormanni P, Camilloni C, Fariselli P, Vendruscolo M (2015). The s2D method: simultaneous sequence-based prediction of the statistical populations of ordered and disordered regions in proteins. J Mol Biol.

[49] Liu Z (2016). Bivalent Copper Ions Promote Fibrillar Aggregation of KCTD1 and Induce Cytotoxicity. Sci Rep.

[50] Biancalana M, Koide S (2010). Molecular mechanism of Thioflavin-T binding to amyloid fibrils. Biochim Biophys Acta.

[51] Smaldone G (2018). The essential player in adipogenesis GRP78 is a novel KCTD15 interactor. Int J Biol Macromol.

[52] Milunsky JM (2008). TFAP2A mutations result in branchio-oculo-facial syndrome. Am J Hum Genet.

[53] Ano Bom AP (2012). Mutant p53 aggregates into prion-like amyloid oligomers and fibrils: implications for cancer. J Biol Chem.

[54] Sengupta, S., Maji, S. K. & Ghosh, S. K. Evidence of a prion-like transmission of p53 amyloid in Saccharomyces cerevisiae. *Mol Cell Biol*, 10.1128/MCB.00118-17 (2017). 10.1128/MCB.00118-17 PMC5574041 28630281

[55] Winklhofer KF, Tatzelt J, Haass C (2008). The two faces of protein misfolding: gain- and loss-of-function in neurodegenerative diseases. Embo J.

[56] Di Natale C (2015). Nucleophosmin contains amyloidogenic regions that are able to form toxic aggregates under physiological conditions. FASEB journal: official publication of the Federation of American Societies for Experimental Biology.

[57] Silva JL, De Moura Gallo CV, Costa DC, Rangel LP (2014). Prion-like aggregation of mutant p53 in cancer. Trends Biochem Sci.

