# Supplementary material for: Molecular basis of the scalp-ear-nipple syndrome unraveled by the characterization of disease-causing KCTD1 mutants
Source: Sci Rep. 2019 Jul 19;9:10519. doi: 10.1038/s41598-019-46911-4 (PMC6642198; doi:10.1038/s41598-019-46911-4)
Supplement: Supplementary file 1 — Supplementary Material [file 41598_2019_46911_MOESM1_ESM.pdf]

Supplementary Material

Molecular basis of the scalp-ear-nipple syndrome unraveled by the characterization of disease-causing KCTD1 mutants.

Giovanni Smaldone[a], Nicole Balasco[b], Luciano Pirone[b], Daniela Caruso[b,c], Sonia Di Gaetano[b], Emilia Maria Pedone[b], and Luigi Vitagliano[b,*]

[a]IRCCS SDN, Via Gianturco 113, 80143 Napoli, Italy

[b]Institute of Biostructures and Bioimaging, CNR, Via Mezzocannone 16, 80134 Napoli, Italy

[c]Università degli Studi della Campania "Luigi Vanvitelli", Viale Abramo Lincoln 5, 81100 Caserta, Italy

*corresponding author: Luigi Vitagliano, luigi.vitagliano@unina.it, 0039 081 2534506

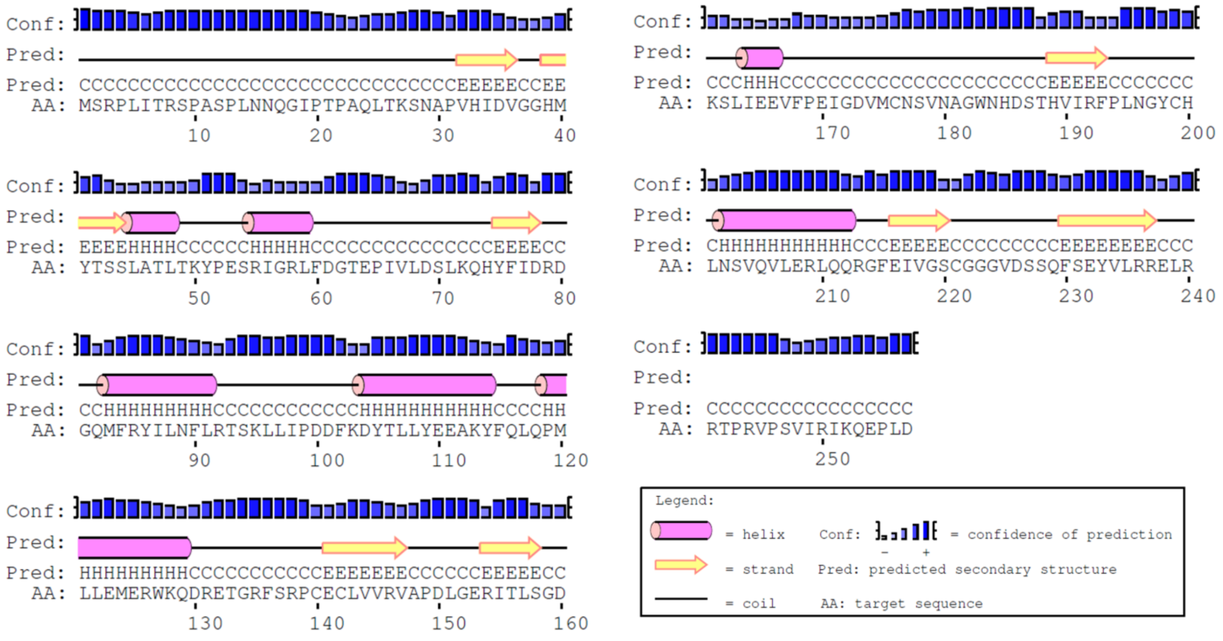

A

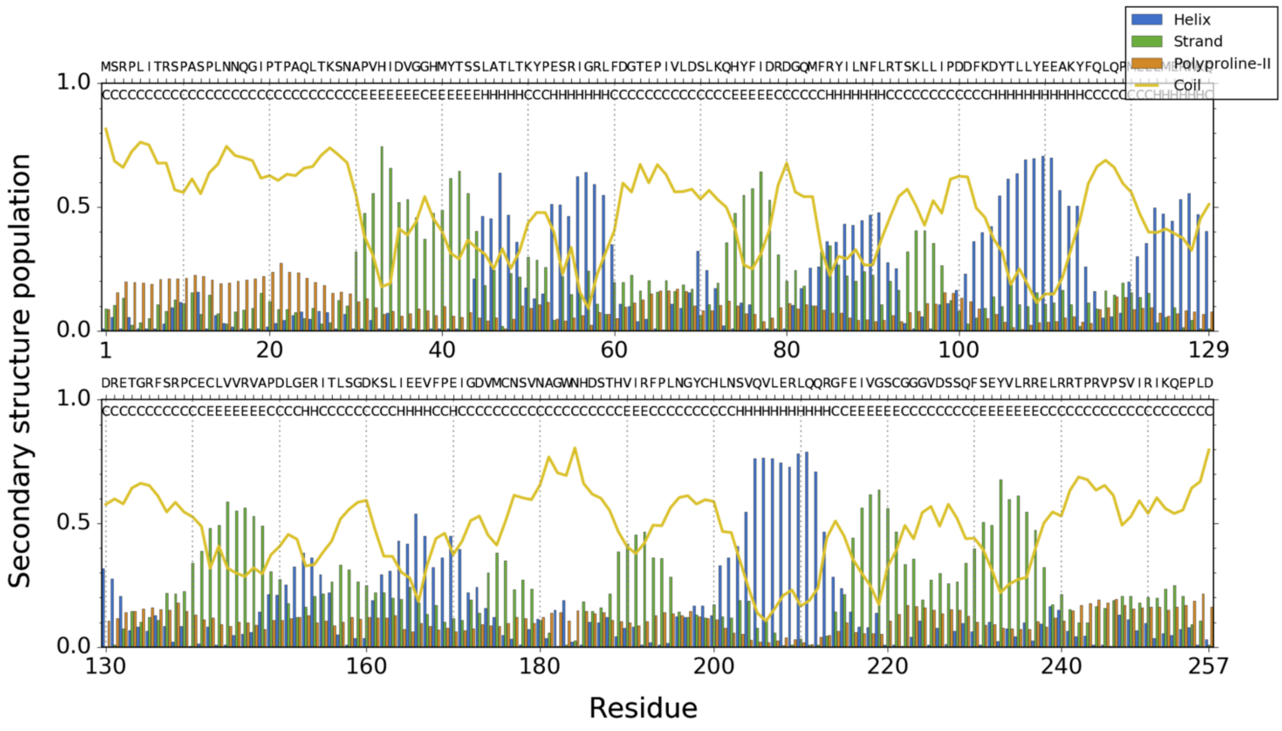

B

Figure S1: Secondary structure prediction of KCTD1 sequence computed by using Psipred server [47] (A) and Vendruscolo Lab. Software [48] (B).

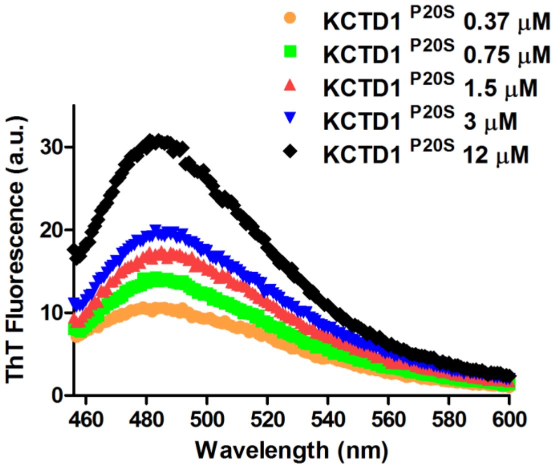

A

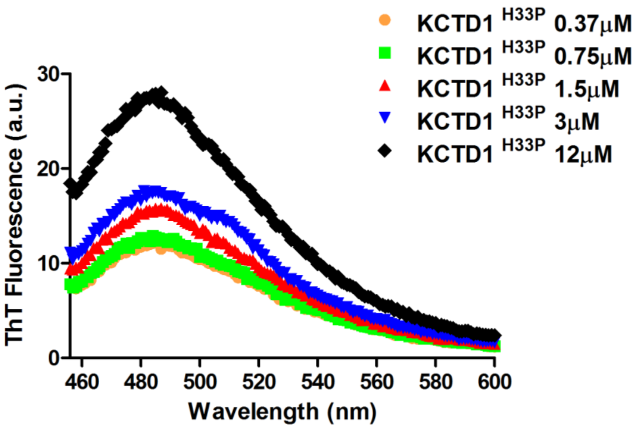

B

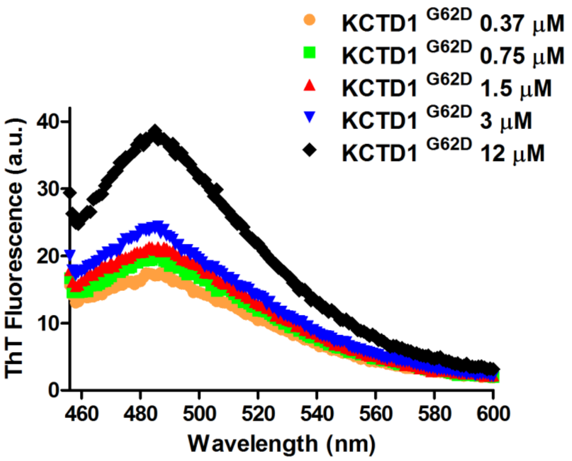

C

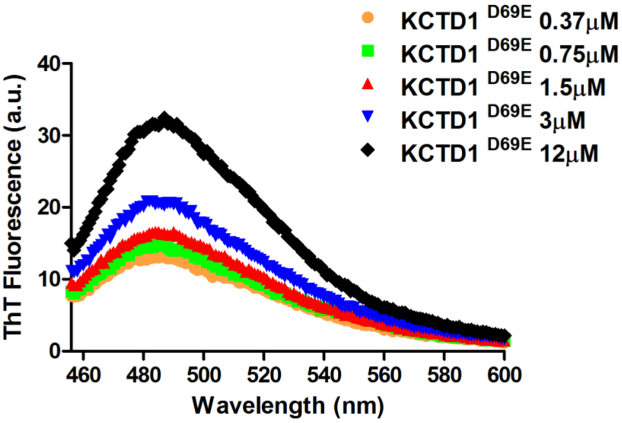

D

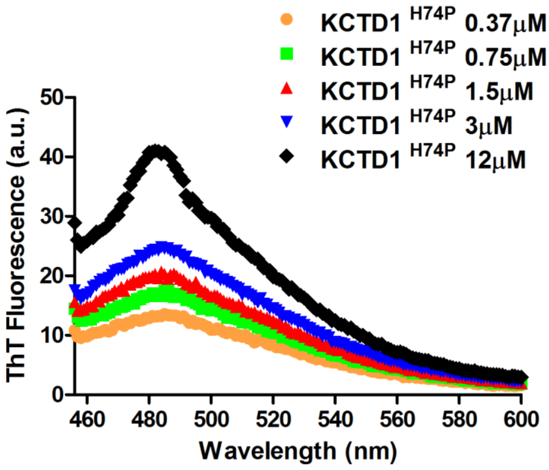

E

Figure S2. Thioflavin T Fluorescence assay: emission spectra obtained by adding a ThT solution (50μM) to KCTD1^{P20S} (A), KCTD1^{H33P} (B), KCTD1^{G62D} (C), KCTD1^{D69E} (D), and KCTD1^{H74P} (E) at protein concentrations ranging from 0.37 μM to 12 μM.

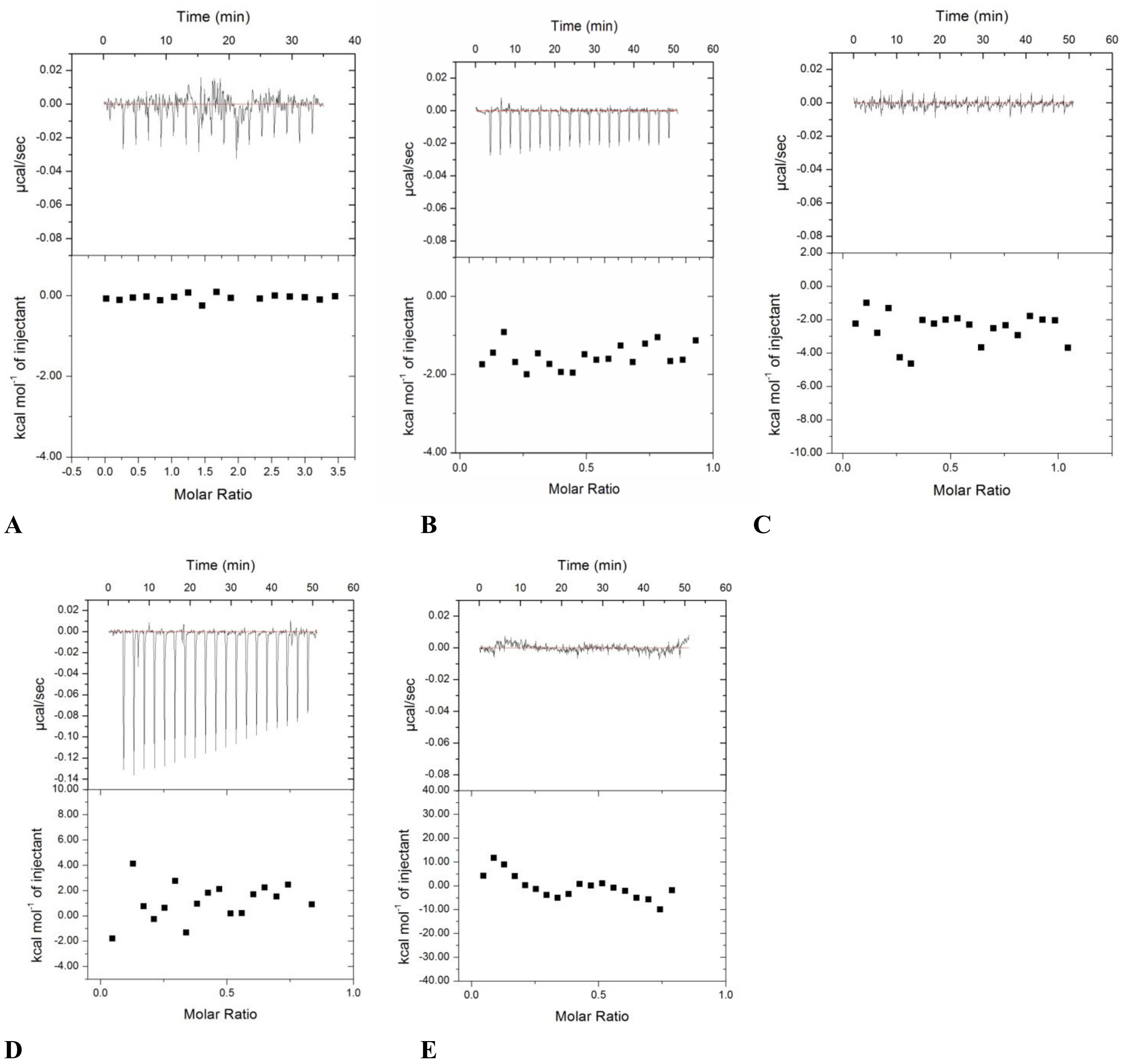

Figure S3: Evaluation of the binding of KCTD1 SEN related mutants $KCTD1^{P20S}$ (A), $KCTD1^{H33P}$ (B), $KCTD1^{G62D}$ (C), $KCTD1^{D69E}$ (D), $KCTD1^{H74P}$ (E) to AP-2α^{NTD} by Isothermal Titration Calorimetry. ITC experiments were performed by titrating KCTD1 variants (72 µM-expressed as pentamer) into a solution of AP-2α^{NTD} (7 µM). Top and bottom panels report raw and integrated data, respectively.

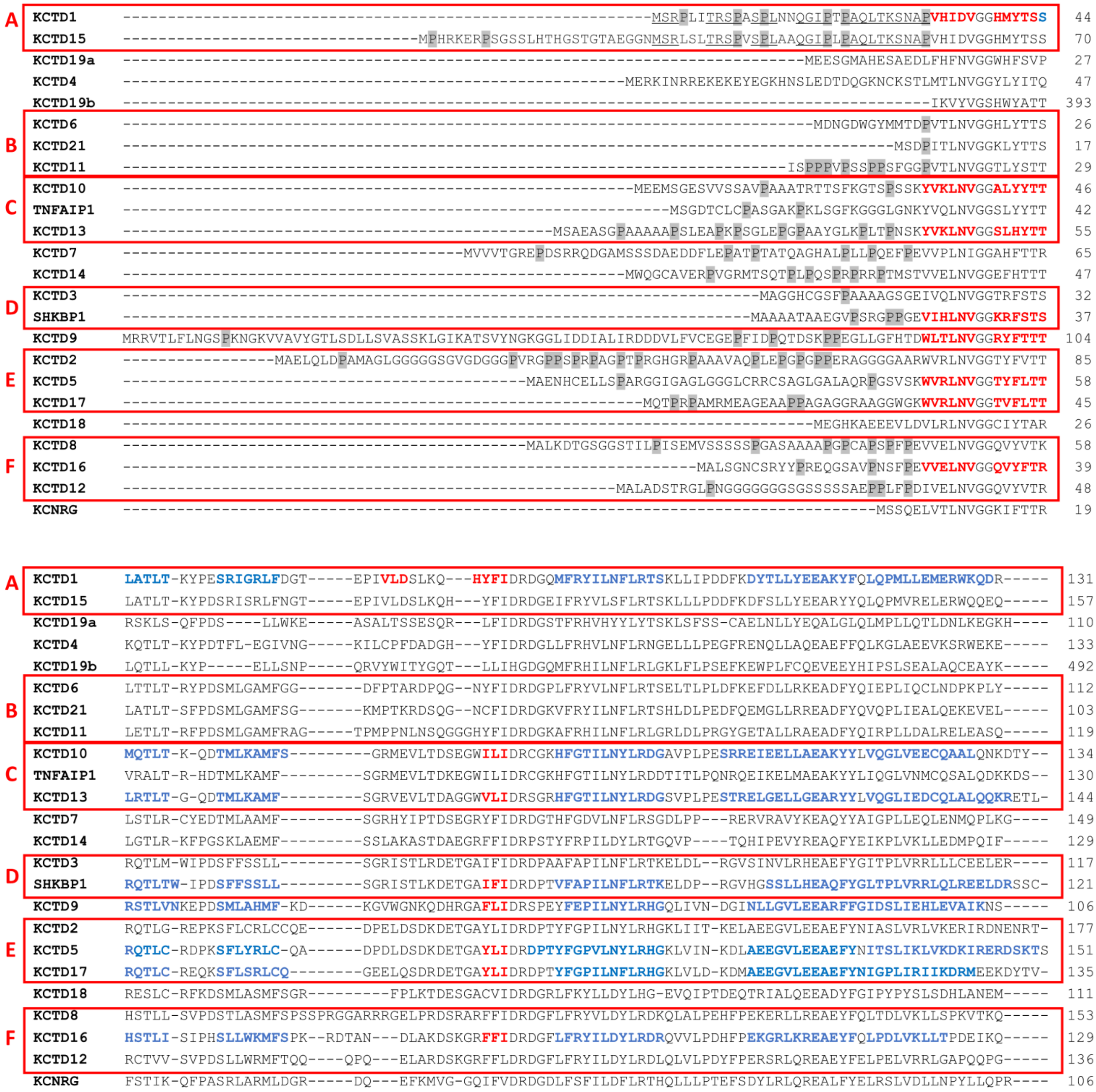

Figure S4: Sequence alignment of members of the KCTD family grouped into the clades defined by Skoblov et al. [24]. The proline residues of the pre-BTB regions are highlighted in grey. Secondary structure elements of the KCTD proteins whose crystal structure has been reported are colored in blue (α-helices) and red (β-sheets). KCTD20 and BTBD10 are not reported as they present a rather low sequence identity with the other members of the family.

Table S1. SEC data of KCTD1, KCTD1^{BTB}, and KCTD1^{CTD}.

Protein	Retention Volume (mL)	Calculated Molecular weight (kDa)	Theoretical Molecular weight of the monomer (kDa)	Ratio
**KCTD1**	12.5	131.22	29.46	4.5
**KCTD1^{BTB}**	14.6	60.81	13.20	4.6
**KCTD1^{CTD}**	14.5	63.10	14.04	4.5

Table S2: Percentages of sequence identity, computed on the BTB domain and on the pre-BTB portion, between human KCTD1 and its orthologs isolated from five species: 4 different classes of the Chordata phylum (*Gallus gallus, Danio rerio, Mouse, Alligator mississippiensis*) and 1 of the Nematoda phylum (*Caenorhabditis elegans*).

KCTD1	*Mouse*	*Caenorhabditis elegans*	*Danio rerio*	*Alligator mississippiensis*	*Gallus gallus*
BTB	100	95.0	89.1	97.0	97.0
Pre-BTB	100	96.7	61.3	100	100

Table S3. Oligonucleotides used for the preparation of KCTD1 proteins. NcoI and XhoI restriction site used for KCTD1^{CTD} cloning are underlined.

Protein	Oligonucleotides
**KCTD1^{CTD}**	Fw: CATGCCATGGGGACCGGTCGTTTTAGCCGTC<br>Rev: CATGCTCGAGTTAATCCAGCGGTTCTTGTT
**KCTD1^{P20S}**	Fw: CTGAATAATCAGGGTATTTCGACACCGGCACAGCTGAC<br>Rev: GTCAGCTGTGCCGGTGTCGAAATACCCTGATTATTCAG
**KCTD1^{H33P}**	Fw: CAAAAGCAATGCACCGGTTCCTATTGATGTTGGTGGTCATATG<br>Rev: CATATGACCACCAACATCAATAGGAACCGGTGCATTGCTTTTG
**KCTD1^{G62D}**	Fw: GGTCGTCTGTTTGATGACACCGAACCGATTGTTCTG<br>Rev: CAGAACAATCGGTTCGGTGTCATCAAACAGACGACC
**KCTD1^{D69E}**	Fw: GAACCGATTGTTCTGGAAAGCCTGAAACAGCACTATTTC<br>Rev: GAAATAGTGCTGTTTCAGGCTTTCCAGAACAATCGGTTC
**KCTD1^{H74P}**	Fw: CTGGATAGCCTGAAACAGCCCTATTTCATTGATCGTGATG<br>Rev: CATCACGATCAATGAAATAGGGCTGTTTCAGGCTATCCAG
